# Phytogenic feed additive mitigates necrotic enteritis-associated gut damage and performance loss in broilers

**DOI:** 10.1016/j.psj.2026.106592

**Published:** 2026-02-03

**Authors:** Most Khairunnesa, Alip Kumar, Shu-Biao Wu, Sharmin Akter, Reza Barekatain, Kowsig Palanisamy, Kosar Gharib-Naseri

**Affiliations:** a School of Environmental and Rural Science, University of New England, Armidale, NSW 2351, Australia; b South Australian Research and Development Institute, Roseworthy Campus, Roseworthy, SA 5371, Australia; c EW Nutrition, Hogenbögen 1, 49429 Visbek, Germany

**Keywords:** Necrotic enteritis, Performance, Broiler, Phytogenic blend, Histomorphology, Inflammation, Mortality

## Abstract

Necrotic enteritis (**NE**) remains a major challenge in broiler production following the withdrawal of antibiotic growth promoters. In response, phytogenic feed additives (**PFA**) have emerged as promising alternatives. This study investigated the efficacy of a phytogenic blend (**PHB**) containing thymol, oleic acid, capric acid, and refined sunflower fat in mitigating NE-associated compromise in performance, gut health, liveability, oxidative stress, and excreta biomarkers of intestinal inflammation in broilers. A total of 450 Cobb 500 D-old chicks were randomly allocated to three dietary groups: **NC** (non-challenged control), **CC** (NE-challenged control), and **PHB** (NE-challenged birds fed 0.01% phytogenic blend), with 10 replicates of 15 birds each. Birds in the CC and PHB groups were orally inoculated with *Eimeria* spp. on d9 and *Clostridium perfringens* at d14 to induce NE. Birds were fed starter (d0-10), grower (d10-24), and finisher (d24-35) diets. During the starter phase (d0-10), birds fed PHB had significantly lower body weight gain (**BWG**) (*P* < 0.05) and higher FCR (*P* < 0.05) compared to the controls. NE challenge significantly (*P* < 0.05) reduced BWG, feed intake (**FI**), and increased FCR during d10-24, d10-35, and d0-35. During d10-35, PHB supplementation significantly improved BWG and FI and reduced FCR (*P* < 0.05) compared with the CC group. While NE challenge impaired gut morphology by reducing villus height (**VH**), VH/crypt depth (**CD**), and increasing intestinal lesion scores (*P* < 0.05), birds in the PHB group showed reduced duodenal lesions and increased VH/CD (*P* < 0.05) compared to CC birds. Furthermore, NE-related mortality and inflammation markers in excreta, such as calprotectin, fibronectin, and lipocalin-2, were significantly higher (*P* < 0.05) in NE-challenged birds compared to the NC group. Supplementation with PHB shifted liveability, and excreta calprotectin and fibronectin values towards the NC group (*P* > 0.05). These findings suggest that PHB supplementation improves intestinal histomorphology and liveability, reduces NE-related mortality, and alleviates inflammation, which together may contribute to the enhanced performance of the PHB-supplemented birds.

## Introduction

Necrotic enteritis (**NE**), caused by the gram-positive bacterium *Clostridium perfringens*, is a major intestinal disease in poultry, responsible for global economic losses estimated at USD $6 billion annually, according to a decade-old estimate (Wade and Keyburn, 2015). Subclinical NE compromises performance, while clinical NE can result in high mortality (Kaldhusdal et al., 2001; Van Immerseel et al., 2004). Historically, antibiotics were used to prevent and treat NE to maintain production performance and gut health by modulating microbiota, reducing bacterial fermentation, and limiting inflammation (Carlson and Fangman, 2018; Gaskins et al., 2002; Prescott et al., 1978). However, due to rising concerns over antibiotic resistance, the European Union banned the use of antibiotics in animal feed as of January 1, 2006 (Castanon, 2007), and similar restrictions have been introduced in many countries to reduce routine antimicrobial use in animal feeds. The removal of antibiotics from poultry feed allows opportunistic pathogens, such as *C. perfringens*, to proliferate, thereby increasing the prevalence of NE. As a result, non-antibiotic strategies are increasingly being explored to support gut health and production efficiency. This includes probiotics, prebiotics, organic acids, enzymes, phytogenic extracts, bacteriophages, and vaccination (Dahiya et al., 2006). Among these, phytogenic feed additives (**PFA**), natural products derived from plants such as herbs and spices, have gained attention due to consumer preference for natural food production systems. The PFA are generally recognized as safe and are therefore widely utilized in the food industry (Varel, 2002). Studies have demonstrated their efficacy in improving performance and gut health in NE-challenged broilers (Blue et al., 2024; Engberg et al., 2012; Tosi et al., 2013). The phytogenic blend (**PHB**) used in the current study, a type of PFA, is formulated with thymol, oleic acid, refined sunflower fat, and capric acid. Thymol, a phenolic compound, has shown strong antibacterial activity against *C. perfringens in vitro* (Du et al., 2015; Timbermont et al., 2010) and anti-inflammatory properties in a rodent model (Riella et al., 2012). This compound has also been shown to enhance mucosal barrier function and reduce inflammation in *Salmonella Typhimurium*-challenged broilers (Ibrahim et al., 2021). Many studies have shown the benefits of combined carvacrol and thymol on improved performance, antioxidant status, immune responses, and intestinal integrity in broiler chickens (Du et al., 2016; Hashemipour et al., 2013; Tiyaprasertkul et al., 2025). Fatty acids, such as oleic acid, a long-chain monounsaturated fatty acid, and capric acid, a medium-chain fatty acid, are gaining attention as functional feed additives due to their antibacterial, anti-inflammatory, and anticoccidial properties (Borniquel et al., 2010; Desbois and Smith, 2010; Tan and Long, 2012). Their antimicrobial activity is mainly attributed to their ability to increase cell membrane permeability, leading to cell lysis and inhibiting membrane enzymatic activities and nutrient uptake (Desbois and Smith, 2010; Gomez-Osorio et al., 2021; Won et al., 2007; Yoon et al., 2018). Oleic acid has been reported to improve performance, egg quality, and meat quality in poultry (Atoo et al., 2024; Ortiz et al., 2006; Toomer et al., 2021), while blends containing capric acid have been shown to improve performance, mortality, and intestinal health in NE-challenged broilers (Abdelli et al., 2020; Kumar et al., 2021a, 2021b). The PHB was applied to investigate its potential against the NE challenge, as its components have been shown to provide antimicrobial, anti-inflammatory, and antioxidative properties. Together, these actions may suppress *C. perfringens*, reduce intestinal inflammation, and support overall gut health. However, the efficacy of this specific PHB under NE challenge has not been well established. Therefore, it is hypothesized that dietary supplementation with PHB alleviates the adverse effects of NE, supports intestinal integrity, reduces oxidative stress, improves performance, and enhances overall health and productivity in broiler chickens. To test the hypothesis, the objective of the present study was to investigate the impact of PHB on growth performance, liveability, gut lesions, gut morphology, and the inflammatory and oxidative stress defense capacities of birds challenged with NE.

## Materials and methods

### Animal ethics

The experimental procedures conducted in this study were approved by the Animal Ethics Committee of the University of New England, Armidale, NSW 2351, Australia (Approval number: ARA23-094). All procedures, accredited by the Australian Bureau of Animal Health, adhered to the Australian Code for the Care and Use of Animals for Scientific Purposes (NHMRC, 2013).

### Experimental procedures, design, and diets

A total of 450 d-old mixed-sex Cobb 500 broiler chicks were procured from Baiada hatchery in Tamworth, NSW, Australia. Upon arrival, birds were weighed and randomly allocated to 30-floor pens, ensuring uniform initial pen weights across experimental treatments. The study was arranged in a completely randomized design comprising three treatments, each with ten replicates of 15 birds per pen. The treatments were: 1) non-challenge control group without additives (**NC**); 2) NE challenge group without additives (**CC**) and 3) NE challenge group supplemented with 0.01% phytogenic blend **(PHB**). Birds were reared in an environmentally controlled house where softwood shavings were used as bedding material to a depth of approximately 8 cm. One tube feeder and three nipple drinkers were used in each pen to allow *ad libitum* feeding and drinking. Temperature, humidity, and lighting schedules were maintained according to Cobb 500 management guidelines (Cobb 500, 2022b). The room temperature was initially kept at 33°C for the first two days of the trial and subsequently reduced by 2°C every two days until it reached 22°C by d21. Birds were fed a corn-soybean-sorghum-wheat-based diet supplemented with phytase at 1000 FTU/kg (100 g/t), considering matrix values, and xylanase was added at 100 g/t. Feed was provided as crumble form during the starter (d0-10) phase, and pellets during the grower (d10-24) and finisher (d24-35) phases. The nutrient composition of the feed ingredients was analyzed using near-infrared spectroscopy (NIRS, AminoNIR Evonik Amino Prox, Essen, Germany) before diet formulation. Diets were formulated following the nutrient specifications recommended for Cobb 500 broilers (Cobb 500, 2022a). The composition and nutrient content of the diets for each phase are presented in Table 1.

**Table 1:** Diet composition (as-fed basis, %) and calculated nutrients.

Ingredients (%)	Starter (d0-10)	Grower (d10-24)	Finisher (d24-35)
Corn	34.0	34.0	39.1
Soybean meal	34.0	28.5	22.8
Sorghum	15.2	20.2	20.3
Wheat	12.5	12.5	12.6
Dicalcium Phosphate	1.10	0.414	0.315
Limestone	1.09	1.10	1.04
Canola oil	0.300	0.867	1.280
1Mineral Premix	0.100	0.100	0.100
2Vitamin Premix	0.090	0.090	0.090
DL-methionine	0.320	0.310	0.306
L-lysine HCl 78.4	0.305	0.346	0.410
Salt	0.216	0.230	0.230
L-threonine	0.130	0.120	0.126
Choline Cl 70%	0.082	0.110	0.139
L-Arginine HCl	0.066	0.119	0.230
L-Valine	0.040	0.049	0.083
Na bicarbonate	0.020	0.020	0.024
4Xylanase	0.010	0.010	0.010
3Phytase	0.010	0.010	0.010
5Sand	0.419	0.960	0.871
6Calculated nutrients (%, otherwise as indicated)			
Dry Matter	89.9	89.9	89.9
AME (kcal/kg)	2920	3010	3100
Crude protein	22.5	20.5	18.5
Crude fat	2.55	3.24	3.75
Crude fiber	3.00	2.90	2.85
Digestible Arg	1.36	1.25	1.18
Digestible Lys	1.26	1.16	1.08
Digestible Met	0.636	0.600	0.570
Digestible Meth+Cyst	0.940	0.881	0.826
Digestible Trp	0.286	0.257	0.226
Digestible Iso	0.855	0.765	0.668
Digestible Thr	0.867	0.780	0.705
Digestible Val	0.959	0.880	0.820
Calcium	0.960	0.800	0.740
Available Phosphorus	0.540	0.400	0.370
Sodium	0.170	0.170	0.170
Potassium	1.041	0.938	0.839
Chloride	0.243	0.265	0.284
Choline mg/kg	1710	1717	1715
Linoleic 18:2	0.913	1.02	1.15

AME=apparent metabolizable energy.

1 Trace mineral concentrate supplied per kilogram of diet: Cu (sulfate), 16 mg; Fe (sulfate), 40 mg; I (iodide), 1.25 mg; Se (selenate), 0.3 mg; Mn (sulfate and oxide), 120 mg; Zn (sulfate and oxide), 100 mg; cereal-based carrier, 128 mg; mineral oil, 3.75 mg.

2 Vitamin premix per kg diet: vitamin A, 12 MIU; vitamin D, 5 MIU; vitamin E, 75 mg; vitamin K, 3 mg; nicotinic acid, 55 mg; pantothenic acid, 13 mg; folic acid, 2 mg; riboflavin, 8 mg; cyanocobalamin, 0.016 mg; biotin, 0.25 mg; pyridoxine, 5 mg; thiamine, 3 mg; antioxidant, 50 mg.

3 Phytase: Axtra phytase gold, 1000 FTU/kg of diet.

4 Xylanase: Rovabio (Addisseo) 100g/t.

5 Sand was replaced with the required amount of PHB and added to the top.

6 Nutrient contents were measured using near-infrared spectroscopy (NIRS, Evonik Amino Prox, Germany).

### NE challenge

The present study utilized a previously established NE challenge model, described by Wu et al. (2014) and Rodgers et al. (2015), using *Eimeria* spp. vaccine strains and *C. perfringens* to induce NE. Briefly, birds in the challenge groups were orally gavaged with 1 mL of a solution containing *Eimeria* spp. oocysts, including 5000 *Eimeria acervulina* and *E. maxima*, and 2500 *E. brunetti* oocysts (Eimeria Pty Ltd., Ringwood, VIC, Australia), on d9. On d14, challenged birds received 1 mL of *C. perfringens* (EHE-NE18) suspension, containing approximately 10^8^ CFU (CSIRO Livestock, Geelong, VIC, Australia), via oral gavage. Meanwhile, birds in the non-challenged group were administered 1 mL of phosphate-buffered saline (**PBS**) on d9 and 1 mL of sterile thioglycolate broth on d14. Necropsies were performed on birds that died following the NE challenge to determine the cause of death. All mortalities were documented, along with the bird's sex and weight throughout the trial.

### Performance

Pen body weight (**BW**) and feed intake (**FI**) were recorded on d10, d24, and d35, and body weight gain (**BWG**), FI, and feed conversion ratio (**FCR**) were calculated for each feeding phase and for the overall period. Daily mortality and body weight of dead birds were recorded, and the FCR was adjusted accordingly. The FI was calculated using the dry matter (**DM**) content of the feed, including both feed-in and feed-out, and the FCR was also determined on a DM basis. Thereafter, both FI and FCR values are reported based on an 88% DM basis (Noblet et al., 2015; Khairunnesa et al., 2025). Between d14-20, if the death was determined as NE-related, it was separately calculated and reported as a percentage of NE-related mortality. On d35, individual BW of birds was recorded to calculate flock uniformity. The coefficient of variation (**CV**) of the BW of birds for each treatment group was calculated to determine the flock uniformity. On d35, all the remaining birds were dissected, and sex was determined by visual inspection of the gonads. European production efficiency factor **(EPEF)** was calculated for phases d10-35 and d0-35 using the following formula,

EPEF= ((Liveability (%) × Average daily gain (g) ÷ (FCR × Age in days)) × 100

### Sample collection, preparation, and lesion scoring

On d16, 2 birds were randomly selected, weighed, and stunned (JF poultry equipment, Weltevreden Park, South Africa). Blood samples were collected individually from each of the two birds via jugular vein using clot-activator vacutainers following a small incision in the neck before decapitation. Collected blood specimens were allowed to clot for 3 hours at room temperature, then centrifuged at 3000 × *g* for 10 minutes. The resulting serum was promptly stored at −20°C until further analysis for total antioxidant capacity (TAC) and malondialdehyde (MDA) measurements. Duodenum and jejunum were excised for lesion scoring. Before lesion scoring, a 5-cm segment of jejunal tissue from one bird per pen was excised, flushed with phosphate-buffered saline (**PBS**), and preserved in 10% neutral buffered formalin (**NBF**) for histomorphology. The duodenum and jejunum were visually examined for NE lesions and scored based on a previously established scoring system (Keyburn et al., 2006; Shojadoost et al., 2013), with lesion scores ranging from 0 to 6, where 0 indicated no lesions and 6 represented the most severe and acute intestinal lesions. Lesion scoring was conducted independently by two experienced personnel who were blinded to the treatment groups to minimize bias. On d28, the same procedures for TAC and MDA analysis were followed to collect, process, and store blood samples. On d16, fresh pooled excreta were collected from each pen and stored at −80°C for excreta biomarker analysis.

### Jejunal histomorphology

For histomorphology analysis, 5 µm jejunal tissue sections were prepared and stained using the standard Haematoxylin and Eosin (H&E) protocol described by Golder et al. (2011). The slides were scanned with a Hamamatsu Nano Zoomer 2.0 RS Slide Scanner (Hamamatsu Photonics K.K., Higashi-ku, Hamamatsu, Japan), and histological measurements were conducted using NDP. View 2.5 software. Parameters such as villus height (**VH**), crypt depth (**CD**), and villus width (**VW**) were measured from 10 randomly selected villi and their associated crypts on a single section per bird. Apparent villus surface area (**VSA**) was calculated using the formula, VSA = 2π (VW/2) (VH), previously described by Sakamoto et al. (2000).

### Malondialdehyde and total antioxidant capacity analysis

Commercial ELISA kits for chicken malondialdehyde (MBS741384) and total antioxidant capacity (Oxiselect, STA-360) assay kits were sourced from MyBioSource (San Diego, CA) and Cell Biolabs, INC. (San Diego, CA, USA), respectively. The assays were carried out according to the manufacturer’s instructions. Each blank and standard solution was replicated twice on each plate, and samples were assayed in duplicates. Optical densities for all assays were determined using a microplate reader (SPECTROstar Nano, BMG LABTECH Pty. Ltd, Australia).

### Excreta sample preparation

Excreta samples collected on d16 and stored at −80°C were thawed and diluted (1:10) with PBS (1.5g: 15 ml PBS) as per the procedures described by Barekatain et al. (2020). The samples were then thoroughly mixed and centrifuged at 1,500 × *g* for 20 min at 4°C (Centrifuge 5810 R, Hamburg, Germany). Aliquots of the supernatants for each sample were then obtained and kept at −80°C until further assays.

### ELISA assays for biomarker analysis

Commercial ELISA kits for chicken calprotectin (MBS7606348), fibronectin (MBS1603106), intestinal alkaline phosphatase (MBS734160), and lipocalin-2 (MBS005459) were sourced from MyBioSource (San Diego, CA). All assays were carried out according to the manufacturer’s instructions. Each blank and standard solution was included on each plate in triplicates, and all samples were assayed in duplicates. Optical densities for all assays were determined using a microplate reader (Bio-Rad Benchmarch Plus^TM^, CA, USA).

### Statistical analysis

All the data collected in this study were tested for normal distribution before statistical analysis. The data were analyzed using JMP 18.0 (SAS Institute, Cary, NC, USA). Differences among treatment means were assessed using one-way analysis of variance (ANOVA) and followed by Tukey's test for pairwise comparisons. Female % was included as a covariate for performance data analysis when it was significant (England et al., 2023). Intestinal lesion score and NE-related mortality data were analyzed using the non-parametric Kruskal-Wallis (Wilcoxon) test. Differences between means were considered statistically significant when the P-value was < 0.05 and were considered to show a tendency towards significance with 0.05 < *P* < 0.10. In addition, the term ‘shifting’ describes outcomes showing responses of a treatment group, leading to the value being intermediate between positive and negative groups, without a significant difference from either if the positive and negative groups were significantly different (Sumon et al., 2025).

## Results

### Growth performance

Table 2 represents the effects of NE challenge and dietary PHB supplementation on broiler growth performance across different feeding phases. In the starter phase (d0 −10), birds in the NC and CC groups had higher BWG (*P* < 0.05) and lower FCR (*P* < 0.05) compared to the PHB birds. During the grower phase (d10-24), NE challenge significantly reduced FI and BWG (*P* < 0.05) while FCR increased (*P* < 0.05) in the CC group compared to the NC group. The PHB group did not (*P* > 0.05) show a difference for these parameters compared to CC group. At the finisher phase (d24-35), no significant differences (*P* > 0.05) were observed among the treatments. However, when these two phases were combined (d10-35), the PHB treatment significantly improved BWG, FI, and FCR (*P* < 0.05) compared to the CC group. Additionally, during the overall study period (d0-35), PHB supplementation significantly increased FI (*P* < 0.05) compared to the CC group, with no difference from the NC (*P* > 0.05).

**Table 2:** Effect of phytogenic blend on the performance of birds under necrotic enteritis challenge.

Treatments	(d0-10)			(d10-24)			(d24-35)			(d10-35)			(d0-35)		
	^1^FI (g)	BWG (g)	FCR	FI (g)	BWG (g)	FCR	FI (g)	BWG (g)	FCR	FI (g)	BWG (g)	FCR	FI (g)	BWG (g)	FCR
^1^NC	285^a^	237^a^	1.205^b^	1535^a^	1139^a^	1.349^b^	2040	1249	1.635	3538^a^	2390^a^	1.480^c^	3803^a^	2629^a^	1.447^b^
CC	276^ab^	234^a^	1.183^b^	1333^b^	854^b^	1.558^a^	1991	1230	1.619	3298^b^	2081^c^	1.586^a^	3497^b^	2312^b^	1.513^a^
PHB	275^b^	219^b^	1.256^a^	1364^b^	897^b^	1.522^a^	2081	1302	1.599	3432^a^	2200^b^	1.560^b^	3678^a^	2419^b^	1.520^a^
^2^SEM	3	3	0.011	14	14	0.011	38	25	0.011	42	32	0.011	45	33	0.011
*P*-value	0.021	<0.001	<0.001	<.0001	<0.0001	<0.0001	0.279	0.122	0.157	0.003	<.0001	<.0001	0.0004	<.0001	<.0001

BWG, body weight gain; FI, feed intake; FCR, feed conversion ratio. ^1^NC, non-challenged control; CC, challenged control; PHB, NE-challenge birds fed 0.01% phytogenic blend. ^a-b^ values within a column with no common superscripts differ significantly (*P* < 0.05). ^2^SEM, standard error of mean. FI and FCR values were standardized for 88% dry matter.

### Liveability, flock uniformity, and European production efficiency factor (EPEF)

Table 3 summarizes the results of liveability, flock uniformity, and EPEF. The NE challenge significantly decreased overall (d0-35) liveability (*P* < 0.05) compared to the NC group. The PHB supplementation shifted liveability (86.0%) towards NC (89.3%) from the CC (70.7%) group, showing no difference from NC or CC (*P* > 0.05).

**Table 3:** Effect of phytogenic blend on liveability, flock uniformity, and European production efficiency factor (EPEF) of birds challenged with necrotic enteritis.

Parameters	^1^Treatments				
	NC	CC	PHB	^2^SEM	*P*-value
Liveability, % d0-35	89.3^a^	70.7^b^	86.0^ab^	4.8	0.024
Flock uniformity, CV %	12	13.9	12.4		
EPEF, d10-35	587^a^	404^c^	487^b^	23	<0.0001
EPEF, d0-35	474^a^	335^b^	397^b^	18	<0.0001

CV= coefficient of variation. ^1^NC, non-challenged control; CC, challenged control; PHB, NE-challenge birds fed 0.01% phytogenic blend. ^2^EPEF, European production efficiency factor. ^a-c^ values within a column with no common superscripts differ significantly (*P* < 0.05). ^2^SEM, standard error of mean.

In terms of flock uniformity, the CV of BW followed the order NC < PHB < CC. The lowest CV was observed in NC (12%), followed by PHB (12.4%) and CC (13.9%).

Similarly, the NE challenge significantly reduced EPEF (*P* < 0.05) during both d10-35 and d0-35. However, PHB supplementation significantly improved EPEF during d10–35 (*P* < 0.05), although the effects did not persist over the entire period (*P* > 0.05).

### NE-related mortality

**Fig. 1:**
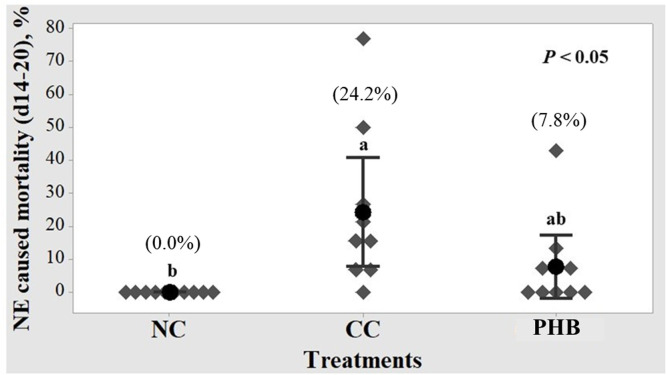
Effect of phytogenic blend on necrotic enteritis (NE) related mortality (d14-20). NC, non-challenge control; CC, challenged control; PHB, NE-challenged birds fed 0.01% phytogenic blend; ^a-b^values within a column with no common superscripts differ significantly (*P* < 0.05).

As shown in Fig. 1, NE-related mortality differed significantly among treatment groups (*P* < 0.05). The CC group had the highest mortality (24.2%), whereas no mortality was observed in the NC group. Interestingly, the PHB group showed an intermediate mortality (7.8%), shifting towards NC, with no significant difference from either CC or NC (*P* > 0.05).

### Lesion score

**Fig. 2:**
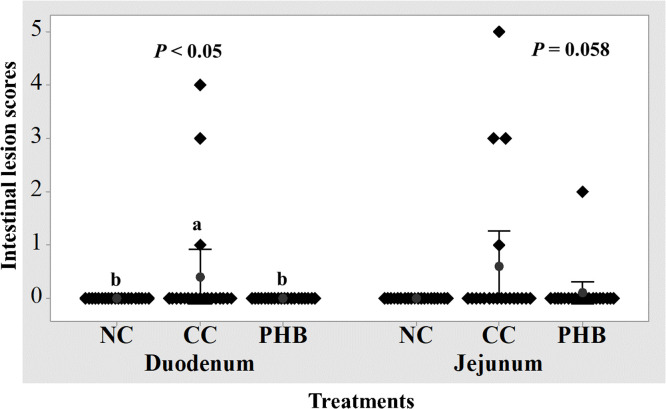
Effect of phytogenic blend (PHB) on d16 intestinal lesions of birds challenged with necrotic enteritis (NE). NC, non-challenged control; CC, challenged control; PHB, NE-challenge birds fed 0.01% phytogenic blend; ^a-b^values within a column with no common superscripts differ significantly (*P* < 0.05).

Fig. 2 shows the d16 intestinal lesion score results. NE challenge significantly increased (*P* < 0.05) duodenal lesions in the CC group compared to the NC birds. Supplementation with PHB significantly reduced (*P* < 0.05) duodenal lesions, showing no difference from the NC group (*P* > 0.05). Jejunal lesion scores showed a trend toward differences among treatments (*P* = 0.058).

### Histomorphology

Table 4 shows the histomorphology results measured at d16. The results indicate that treatments significantly (*P* < 0.05) impacted VH, CD, and the VH/CD ratio. NE challenge significantly reduced VH (*P* < 0.05) and VH/CD (*P* < 0.05) ratio, while increasing CD (*P* < 0.05) in the CC group compared to the NC birds. The PHB group showed a shift in VH (*P* > 0.05) from CC towards the NC birds, showing no difference from CC or NC. Furthermore, the PHB group significantly increased VH/CD (*P* < 0.05) ratio compared to the CC birds.

**Table 4:** Effect of phytogenic blend on d16 jejunal histomorphology of broilers challenged with necrotic enteritis.

Item	Measurements				
	Villus height (µm)	Crypt depth (µm)	Villus width (µm)	Villus surface area (mm^2^)	Villus height/Crypt depth
1NC	1482^a^	191^b^	184	0.864	7.91^a^
CC	1242^b^	331^a^	175	0.689	3.76^c^
PHB	1369^ab^	299^a^	184	0.798	4.67^b^
2SEM	63	12	8	0.059	0.29
*P-*value	0.046	<.0001	0.660	0.133	<.0001

1 NC, non-challenged control; CC, challenged control; PHB, NE-challenge birds fed 0.01% phytogenic blend. ^a-b^values within a column with no common superscripts differ significantly (*P* < 0.05).

2 SEM, standard error of mean.

### Serum total antioxidant capacity (TAC) and malondialdehyde (MDA) (d16 and d28)

Table 5 shows the results of d16 and d28 serum TAC and MDA levels. The NE challenge significantly reduced d16 TAC levels (*P* < 0.05) in the CC group compared to the NC group. The PHB supplementation did not significantly affect TAC (*P* > 0.05) compared to CC. On d16, the MDA level was not different between NC and CC (*P* > 0.05) groups, whereas the PHB group had significantly higher MDA levels than NC (*P* < 0.05) but was not different from CC (*P* > 0.05). On d28, no significant differences (*P* > 0.05) were observed for either TAC or MDA levels among the treatment groups.

**Table 5:** Effect of the phytogenic blend on d16 and d28 serum total antioxidant capacity (TAC) and malondialdehyde (MDA) of broilers challenged with necrotic enteritis.

Treatments	d16 TAC (CRE)	d28 TAC (CRE)	d16 MDA (ng/mL)	d28 MDA (ng/mL)
^1^NC	1640^a^	1497	28.5^b^	43.9
CC	1405^b^	1425	35.7^ab^	44.5
PHB	1401^b^	1403	43.4^a^	43.0
^2^SEM	57	59	3.2	1.6
*P*-value	0.006	0.506	0.009	0.814

CRE, copper reducing equivalent. ^1^NC, non-challenged control; CC, challenged control; PHB, NE-challenge birds fed 0.01% phytogenic blend. ^a-b^values within a column with no common superscripts differ significantly (*P* < 0.05). ^2^SEM, standard error of mean.

### Excreta biomarkers

Table 6 illustrates the inflammation biomarker concentrations in excreta. NE challenge significantly increased calprotectin, fibronectin, and lipocalin-2 (*P* < 0.05) in the CC group compared to the NC, while alkaline phosphatase remained unaffected (*P* > 0.05). The PHB group shifted calprotectin and fibronectin levels towards the NC from the CC group, showing no difference from CC or NC (*P* > 0.05). However, PHB did not show any effect on lipocalin-2 level (*P* > 0.05).

**Table 6:** Effect of phytogenic blend on d16 excreta inflammation biomarkers of birds under necrotic enteritis challenge.

Treatments	Calprotectin (ng/ml)	Fibronectin (µg/ml)	Lipocalin-2 (ng/ml)	Alkaline Phosphatase (ng/ml)
1NC	33.9^b^	79.0^b^	84.4^b^	2.59
CC	36.4^a^	106^a^	118^a^	2.65
PHB	35.2^ab^	96.6^ab^	111^a^	2.54
2SEM	0.5	5.9	5.3	0.26
*P-* value	0.004	0.013	0.0003	0.954

1 NC, non-challenged control; CC, challenged control; PHB, NE-challenge birds fed 0.01% phytogenic blend. ^a-b^values within a column with no common superscripts differ significantly (*P* < 0.05).

2 SEM, standard error of mean.

## Discussion

Necrotic enteritis remains one of the most economically significant intestinal diseases in commercial broiler production (Abd El-Hack et al., 2022), leading to impaired growth performance and increased mortality (Hofacre et al., 2003; Timbermont et al., 2011; Van Immerseel et al., 2004). This study evaluated the potential of PHB supplementation on broiler performance, liveability, intestinal lesions and morphology, flock uniformity, oxidative status, and excreta biomarkers under NE challenge conditions. The successful NE challenge was confirmed by impaired performance, reduced liveability, NE-related mortality, duodenal lesions, altered intestinal histomorphology, and increased excreta inflammation biomarkers. Supplementation of PHB in NE-challenged birds improved d10-35 performance, reduced duodenal lesions, and increased VH/CD ratios, and shifted liveability, NE-related mortality, and excreta biomarkers of inflammation (calprotectin and fibronectin) towards non-challenge levels. Collectively, these findings partially validate the hypothesis that PHB alleviates the adverse effects of NE by improving intestinal histomorphology, reducing NE lesion severity and intestinal inflammation, and thereby contributing to improved performance.

Dietary supplementation of PHB significantly improved BWG, FI, and FCR, as well as EPEF during d10 to d35, and there was an overall (d0-35) 4.6% improvement in BWG, and significantly higher FI (5.2%) of the supplemented birds compared to the CC group. These findings align with previous research showing that PFA improved performance under NE challenge (Abdelli et al., 2020; Blue et al., 2024; Cho et al., 2014; El-Hamid et al., 2024; Kumar et al., 2022). The improved performance observed in PHB-supplemented birds is associated with enhanced gut health, as reflected by significantly lower intestinal lesion scores, a higher VH/CD ratio, and shifts in excreta biomarkers of intestinal inflammation. Birds supplemented with PHB showed a higher VH/CD ratio, a well-established indicator of improved epithelial health and barrier function, thereby enhancing nutrient absorption (Galamatis et al., 2025). Consistent with this, Du et al. (2016) reported improved villus morphology in NE-challenged broilers fed thymol and carvacrol-based essential oils. Similarly, Abdelli et al. (2021) found that microencapsulated thymol improved the VH/CD ratio. This beneficial effect of PHB may arise from the anti-inflammatory actions of thymol and oleic acid, which help protect against mucosal damage and support the integrity of the villus structure, characterized by taller villi and shallower crypts (Ghiselli et al., 2022; Islam et al., 2024; Santa-María et al., 2023). Additionally, PHB supplementation significantly reduced duodenal lesions, indicating its ability to suppress colonization by *Eimeria* and *C. perfringens* in the gut. These findings are consistent with previous studies in broilers using essential oil blends (25% thymol and 25% carvacrol) (Yin et al., 2017), and other combinations like thymol, cinnamaldehyde, and eucalyptus oil (Timbermont et al., 2010), as well as other PFA (Cho et al., 2014), all of which demonstrated reduced gut lesions and improved gut health under NE challenge. The protective effects of PHB are likely due to its individual components. For example, thymol disrupts bacterial membranes and reduces *C. perfringens* colonization (Du et al., 2015; Ghiselli et al., 2022; Timbermont et al., 2010), while fatty acids such as oleic and capric acid contribute to this antimicrobial effect through mechanisms including microbial cell lysis, enzyme activity inhibition, nutrient uptake impairment, and the production of toxic peroxidation and auto-oxidation products (Desbois and Smith, 2010). The observed reduction of duodenal lesions in PHB-supplemented birds may therefore be partially attributable to these antimicrobial mechanisms, which could have limited *C. perfringens* colonization and brought them closer to those seen in the NC birds. Notably, the CC birds showed significant increases in excreta inflammatory protein markers, including calprotectin, a neutrophil protein that signals mucosal inflammation (Foell et al., 2009); fibronectin, an extracellular matrix glycoprotein, released during epithelial damage and tissue remodelling (To and Midwood, 2011); lipocalin-2, which is associated with host defense and microbial imbalance (Moschen et al., 2017). The increase in these biomarkers is consistent with previous studies on chickens that have shown intestinal inflammation (Barekatain et al., 2020; Cardoso Dal Pont et al., 2021; De Meyer et al., 2019). Supplementation with PHB shifted these marker levels toward those seen in the NC group. This anti-inflammatory effect aligns with the known properties of thymol (Islam et al., 2024) and oleic acid (Santa-María et al., 2023). Together, these gut-level improvements provide insight into PHB's mode of action in enhancing performance and flock-level gains. Furthermore, PHB supplementation improved flock uniformity and liveability, consistent with a previous study on phytogenic blends under NE challenge (Kumar et al., 2022), reinforcing the plausibility of the PHB effects observed here. Based on the underlying mechanism, improved villus structure enhanced absorptive capacity, coupled with reduced epithelial damage and an inflammatory burden, contributed to stabilizing feed intake and diminishing nutrient partitioning towards immune defense, thereby narrowing inter-bird variability in daily gain and enhancing flock uniformity (Akram et al., 2024; Lundberg et al., 2021). The same enhancements in intestinal function and inflammatory status improve overall liveability by decreasing mortality and lowering the culling rate regardless of NE-related mortality. The combined improvement of performance and liveability resulted in a higher EPEF in PHB-supplemented birds (487) compared to the CC group (404). Therefore, these gut-mediated gains provide a coherent explanation for the higher EPEF observed in PHB-supplemented birds, consistent with previous reports of increased EPEF (Adaszyńska-Skwirzyńska et al., 2025; Perić et al., 2009), given that EPEF is mathematically driven by body weight, FCR, and liveability. Collectively, PHB enhanced performance, reduced duodenal lesions, mitigated mucosal damage and inflammation, and translated these gut-level effects into improved uniformity, liveability, and EPEF. However, although PHB supplementation at 0.01% significantly improved performance and intestinal health during the NE challenge period, PHB supplementation during the starter phase did not show the same trend, where it reduced BWG and increased FCR. Similarly, Gharib-Naseri et al. (2021) observed increased FCR during the starter phase (d0-10) when birds were fed with a high dose (0.5%) buffered formic acid product under the same challenge conditions. Interpretation of negative performance response in the early stage (d0-10) was difficult due to the ongoing physiological and microbial adaptation and the absence of sample collection during this period. Therefore, further studies using different doses are required to explain this observation and elucidate the mechanisms by which PHB affects performance and intestinal health throughout the different growth phases.

In broilers, clinical NE is often associated with higher mortality. In line with this, the present study showed that NE challenge markedly increased mortality, particularly during d14-20, a period consistent with peak NE mortality (Wu et al., 2010). Moreover, the increased mortality observed in this phase confirms that the NE challenge applied in this study induced a clinical rather than a subclinical form of the disease (Swaggerty et al., 2022). Despite the severity of NE, PHB supplementation shifted NE-related mortality by 16.4% toward the non-challenge birds compared to the CC during this critical phase, showing a similar improvement trend to that observed with overall liveability. This shift in mortality may be attributed to the ability of PHB to mitigate gut damage and reduce pathogen load, as evidenced by lower duodenal lesion scores, higher VH/CD, and reduced excreta markers of inflammation observed in this study. These findings are consistent with thymol-based improvements previously reported under NE challenge (Abdelli et al., 2020; Dunlop et al., 2016). Overall, PHB supplementation reduced peak NE-related mortality likely by restoring mucosal structure, reducing intestinal lesions, and the burden of gut inflammation.

Oxidative stress is a common consequence of NE, often resulting from excessive intestinal inflammation and tissue damage (Lauridsen, 2019). In this study, NE-challenged birds exhibited significantly lower TAC level and a numerical increase in MDA level on d16, reflecting oxidative stress. Despite PHB’s potential benefits on performance and gut health, the response regarding oxidative status defense capacity remained unchanged. The PHB blend contains bioactive compounds with recognized antioxidant activity levels, including thymol (Nagoor Meeran et al., 2017), oleic acid (Santa-María et al., 2023), and capric (Sengupta and Ghosh, 2012). However, in this study, the effects of these constituents may not have been enough to counteract the oxidative damage induced by the severe NE challenge. The discrepancy between our findings and previous reports on positive effects of these compounds on oxidative stress markers may be explained by differences in disease status and severity of the inflammation (Abd El-Hack and Alagawany, 2015; Mašek et al., 2014; Placha et al., 2019). Another reason may be the rapid absorption of thymol in the upper gut, as reported in piglets (Michiels et al., 2008; Van Noten et al., 2020), that may have limited its systemic availability, reducing its impact on circulating oxidative markers. Overall, in the context of a severe NE challenge, PHB did not measurably improve systemic oxidative markers, likely due to challenge severity and limited systemic availability. It might be likely that a higher dosage of PHB would show a significant effect on antioxidant activity when the severity of NE is high. Therefore, it may warrant further investigation on the dosage of PHB under severe NE challenge conditions.

In conclusion, PHB supplementation at 0.01% enhanced growth performance (d10-35), EPEF, gut morphology, and flock uniformity, reduced gut lesions, and shifted liveability and markers of inflammation in broilers challenged with NE. These advantages are probably due to the antimicrobial and anti-inflammatory properties of its phytogenic components. However, its limited impact on early-life performance may be due to suboptimal dosages of the active compounds in this study, which warrants further investigation. Overall, PHB shows potential as a natural feed additive to mitigate the negative effects of NE in broilers. This study provides evidence that phytogenic solution such as PHB supplementation is promising in antibiotic-free poultry systems to promote gut health, reduce disease burden, and support sustainable production.

## CRediT authorship contribution statement

**Most Khairunnesa:** Writing – review & editing, Writing – original draft, Methodology, Investigation, Formal analysis, Data curation. **Alip Kumar:** Writing – review & editing, Supervision, Methodology, Investigation, Data curation. **Shu-Biao Wu:** Writing – review & editing, Supervision, Data curation. **Sharmin Akter:** Methodology, Data curation. **Reza Barekatain:** Writing – review & editing, Methodology. **Kowsig Palanisamy:** Writing – review & editing, Funding acquisition. **Kosar Gharib-Naseri:** Writing – review & editing, Funding acquisition.

## Disclosures

We declare that we have no financial and personal relationships with other people or organizations that can inappropriately influence our work, there is no professional or other personal interest of any nature or kind in any product, service and/or company that could be construed as influencing the content of this paper.
